# Analysis of the burden and trends of communicable diseases in Pacific Island countries from 1990 to 2019

**DOI:** 10.1186/s12889-023-16894-z

**Published:** 2023-10-21

**Authors:** Yan Li, Hao Li, Yi Jiang

**Affiliations:** School of Public Health, Chongqing Medical University, Chongqing, China

**Keywords:** Communicable diseases, Pacific Islands, Disease, Disability-adjusted life years, Incidence

## Abstract

**Background:**

Communicable diseases contribute substantially to morbidity and death rates worldwide, particularly in low-and middle-income countries. Pacific Island countries face unique challenges in addressing these diseases due to their remote locations and limited resources. Understanding the burden and trends of these diseases in this region is crucial for developing effective public health interventions.

**Objective:**

This study aimed to analyze the burden and trends of communicable diseases in Pacific Island countries from 1990 to 2019.

**Methods:**

We utilized data from the 2019 Global Burden of Disease (GBD) study to analyze indicators including incidence, death, and disability-adjusted life years (DALYs). Excel 2016, R 4.2.1, and GraphPad Prism 9 were used to analyze and visualize the data. Joinpoint regression models were used for trend analysis, and the average annual percent change (AAPC) was calculated.

**Results:**

From 1990 to 2019, the standardized incidence rate of communicable diseases in Pacific Island countries showed an upward trend (AAPC = 0.198%, 95% CI = 0.0174 ~ 0.221), while the standardized death rate (AAPC = -1.098%, 95% CI = –1.34 ~ 0.86) and standardized DALY rate (AAPC = -1.008%, 95% CI = -1.187 ~ -0.828) showed downward trends. In 2019, the standardized incidence, death, and DALY rates of communicable diseases were higher among males than among females, but the standardized death and DALY rates among males decreased faster than those among females from 1990 to 2019. There were significant differences in the disease burden among different Pacific Island countries. The Solomon Islands had the highest standardized death rate (363.73/100,000), and Guam had the lowest (50.42/100,000). Papua New Guinea had the highest standardized DALY rate (16,041.14/100,000), and the Cook Islands had the lowest (2,740.13/100,000). In 2019, the main attributable risk factors for communicable disease deaths in Pacific Island countries were child and maternal malnutrition (28.32%), followed by unsafe water, sanitation, and handwashing (27.14%), air pollution (16.11%), and unsafe sex (14.96%). There were considerable geographical variations in risk factors.

**Conclusion:**

The burden of communicable diseases in Pacific Island countries remains high, despite improvements in mortality and disability-adjusted life-year rates over the past few decades. This study provides valuable insights into the burden and trends of communicable diseases in Pacific Island countries from 1990 to 2019. The findings reveal several important insights and highlight the need for targeted public health interventions in the region.

**Supplementary Information:**

The online version contains supplementary material available at 10.1186/s12889-023-16894-z.

## Introduction

Pacific Island countries are some of the most remote regions in the world [1]. Most of these countries have small territories, weak adaptation abilities to natural disasters, severe shortages of funds and technology, and extremely low levels of economic and social development [2]. The per capita GDP of many of these countries is lower than the global average GDP ($11,570). Compared with other developing countries, Pacific Island countries are the most vulnerable and sensitive to the health threats brought about by climate change [3], with the strongest reactions [4] to the health threats posed by climate change, which can include policy development, social action and the level of public concern and anxiety about climate change [5].Waterborne and foodborne diseases caused by extreme weather events, malnutrition resulting from changes in rainfall patterns, food insecurity due to sea level rise, and the spread of vector-borne diseases due to the increase n or proximity of insect vector breeding grounds were more common in flood-prone areas [6]. In Fiji [7], most of the residents live along the coast, and rising sea levels threaten their lives and property. Furthermore, the infiltration of seawater leading to groundwater salinization poses a substantial threat to the availability of potable water for the people of Fiji [8]. Tonga [9] frequently experiences high temperatures, and in recent years, it has experienced several extreme heat events, some of which have led to deaths and health problems. At the same time, due to rising temperatures, Tonga faces a greater risk of transmission of diseases, such as dengue fever and Zika virus [10]. Niue's droughts have affected food and water supplies, leading to health problems and food shortages. As the Sociodemographic Index (SDI) increases [11], the global burden of disease will shift from communicable, maternal, neonatal, and nutritional diseases (which we refer to as communicable diseases) to noncommunicable diseases. Based on the WHO World Health Statistics 2023, it is projected that noncommunicable diseases will contribute to 75% of global deaths by 2022, a considerable increase from 61% in 2000. Conversely, communicable diseases will account for only 18% of deaths, compared to 31% in 2000 [12]. However, the communicable disease burden in Pacific Island countries (29.13%) is far higher than that in North Africa and the Middle East (15.71%) and Southeast Asia (21.46%). Analyzing the burden of communicable diseases in Pacific Island countries from 1990–2019 can help the international community better understand and support sustainable development in Pacific Island countries while also contributing to the development of disease prevention and control and public health strategies worldwide, which is important for achieving sustainable development in global health. In addition, for Pacific Island countries, understanding and addressing these health challenges are important components in achieving sustainable development, and therefore, analyzing trends and risk factors for these diseases can provide an important scientific basis and guidance for Pacific Island countries. This study analyzed the burden and trends of and risk factors for communicable disease in Pacific Island countries from 1990–2019 based on the Global Burden of Disease 2019 (GBD 2019) study.

## Data and methods

### Data sources

The Global Burden of Disease 2019 [13] (GBD 2019) study, known worldwide as the most comprehensive epidemiological study, was the primary source of data used in this study. The GBD 2019 provides a tool to quantify health losses caused by diseases, injuries, and risk factors to estimate death and disability rates related to over 350 diseases and injuries in 204 countries and regions by age and sex. In this study, the relevant indicators of the communicable disease burden in 18 Pacific Island countries (see Appendix Table 1 for details of the 18 countries) were collected from 1990 to 2019 according to the geographic regions defined by the GBD. Diseases were classified based on the International Classification of Diseases, 10th Revision (ICD-10), including diarrheal diseases, HIV/AIDS, sexually transmitted infections, maternal and neonatal diseases, nutritional deficiencies, respiratory infections and tuberculosis, neglected tropical diseases, malaria, and other communicable diseases [14].

Regarding disease risk factors [7], the GBD 2019 classifies disease risk factors into four levels: level one risk factors include behavioral, environmental/occupational, and metabolic risks; level two risk factors include 20 risk or risk clusters; level three risk factors include 52 risk factors or risk clusters; and level four risk factors include 69 specific risk factors. The computation of specific risk factors and aggregates in GBD 2019 resulted in a total of 87 risks or risk clusters [15]. This study focused on ten level two risk factors that were associated with infectious diseases. Specifically, this study examined the influence of these ten level two risk factors on infectious diseases in Pacific Island countries. Of the twenty level two risk factors considered, only ten had a direct connection to infectious diseases (specific level two risk factors are detailed in Appendix Table 2). The ten level two risk factors included air pollution, child and maternal malnutrition, and unsafe sex, among others.

### Statistical methods

Excel 2016 and R 4.2.1 were used for statistical analysis, and GraphPad Prism 9 was used for data visualization. Trends in the standardized incidence rate, death rate, and disability-adjusted life years (DALYs) of communicable diseases in Pacific Island countries between 1990 and 2019 were calculated using the Joinpoint Regression Program 4.9.0. The incidence rate, death rate, and DALYs were age-standardized using the world standard population [16]. The use of average annual percent change (AAPC) allowed for a combination of long-term change, stability, and comparability in describing trends in the burden of communicable diseases. This approach provided a simplified way to interpret data and helped us better understand and assess the trends in the burden of communicable diseases. By calculating the AAPC, we determined whether there was an upward or downward trend in the indicator. An AAPC > 0 indicated an upward trend, whereas an AAPC < 0 indicated a downward trend [17]. In this study, Joinpoint 4.9.0 software was utilized to calculate the AAPC, thus enabling a more accurate analysis of the trends in the burden of disease and providing valuable information for the development of effective prevention and control measures. The significance of each trend change was tested with a significance level of α = 0.05.

## Results

### Disease burden trends of communicable diseases in Pacific Island countries

The burden of disease for communicable diseases in Pacific Island countries from 1990 to 2019 showed an overall downward trend.

**Fig. 1:**
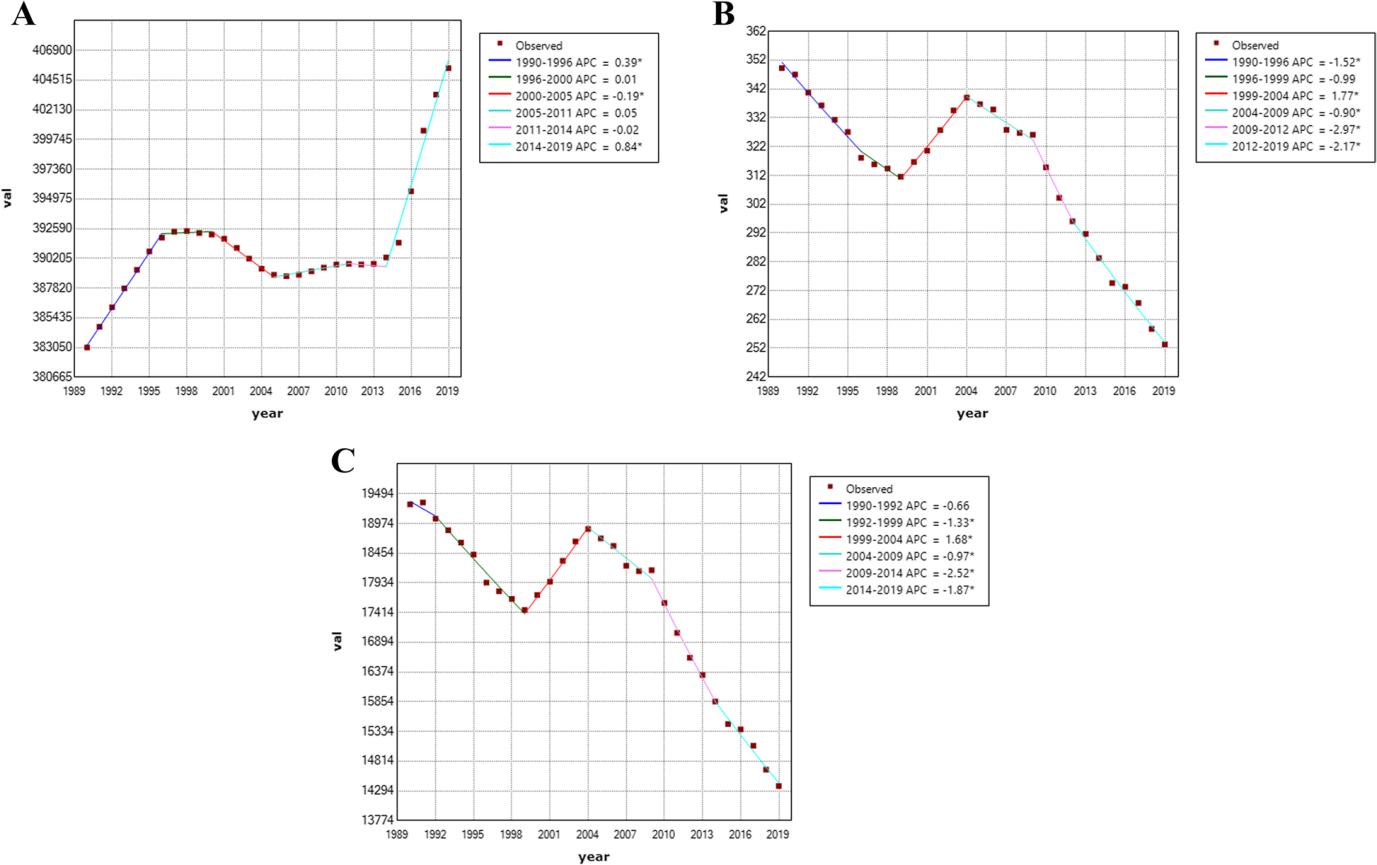
The change trends of communicable diseases. ASIR, ASDR and age-standardized DALY rates from 1990 to 2019. **A** ASIR, age-standardized incidence rate. **B** ASDR, age-standardized death rate. **C** Age-standardized DALY rate. Abbreviations: DALY = disability-adjusted life year

From 1990 to 2019, there was a gradual decline in the overall disease burden of communicable diseases in Pacific Island countries. However, the age-standardized incidence rate showed a slow upward trend, increasing from 383,050/100,000 in 1990 to 405,467/100,000 in 2019, with an average annual percent change (AAPC) of 0.198% (95% CI = 0.0174 ~ 0.221, *P* < 0.001). The growth rate accelerated from 2014 to 2019, with an average annual increase of 0.84% (*P* < 0.001), as shown in Fig. 1 (A).

In contrast to the age-standardized incidence rate, the age-standardized death rate and age-standardized disability-adjusted life year (DALY) rate of communicable diseases in Pacific Island countries from 1990 to 2019 showed a downward trend. The age-standardized death rate declined from 349/100,000 in 1990 to 253/100,000 in 2019, with an AAPC of -1.098% (95% CI = -1.34 ~ -0.86, *P* < 0.001). The fastest decline was from 2009 to 2012, with an average annual decrease of 2.97%, as shown in Fig. 1 (B). The age-standardized DALY rate declined from 19,307/100,000 in 1990 to 14,377/100,000 in 2019, with an AAPC of -1.008% (95% CI = -1.187 ~ -0.828, *P* < 0.001). Similar to the age-standardized death rate, the fastest decline in the age-standardized DALY rate was from 2009 to 2012, with an average annual decrease of 2.52%, as shown in Fig. 1 (C) (refer to Table 1 and Fig. 1 for detailed information on disease burden changes in Pacific Island countries from 1990 to 2019). Figure 1 illustrates the trends in the age-standardized incidence rate, death rate, and DALY rate of communicable diseases in Pacific Island countries from 1990 to 2019. The x-axis represents the years, while the y-axis shows the rates per 100,000 population. The legend displays a color-coded representation of each trend line.

**Table 1:** The disease burden of communicable diseases in Pacific Island countries from 1990 to 2019

	**ASIR(/100, 000)**			**ASDR (/100, 000)**			**Age-standardized DALY rate (/100,000)**		
	1990	2019	AAPC(%)	1990	2019	AAPC(%)	1990	2019	AAPC(%)
**Both**	383,050	405,467	0.198*	349	253	-1.098*	19,307	14,377	-1.008*
**Male**	393,466	423,289	0.257*	367	263	-1.173*	20,040	14,612	-1.083*
**Female**	371,601	386,167	0.137*	330	244	-1.043*	18,518	14,105	-0.956*

(ASIR, age-standardized incidence rate. *ASDR *Age-standardized death rate. age-standardized DALY rate. Abbreviations: *DALY *Disability-adjusted life year.)

^*^ indicates *P* < 0.05

### Disease burden of communicable diseases in Pacific Island countries

#### Sex differences in the disease burden

In 2019, compared to 1990, there was an upward trend in standardized incidence rates for males and females and a downward trend in the age-standardized death rate and DALY rate. In both 1990 and 2019, the age-standardized incidence rate, death rate, and DALY rate of communicable diseases in Pacific Island Countries were higher among males than among females. Moreover, the age-standardized incidence rate of communicable diseases among males increased faster than that among females, with an average annual growth rate 0.12% higher than that among females. However, the decline in the age-standardized death rate and DALY rate of communicable diseases among males was greater than that among females, with an average annual decrease of 1.173% and 1.083%, respectively. Refer to Table 1 for detailed information on sex-specific disease burden in Pacific Island Countries.

#### Age group differences in the disease burden

**Fig. 2:**
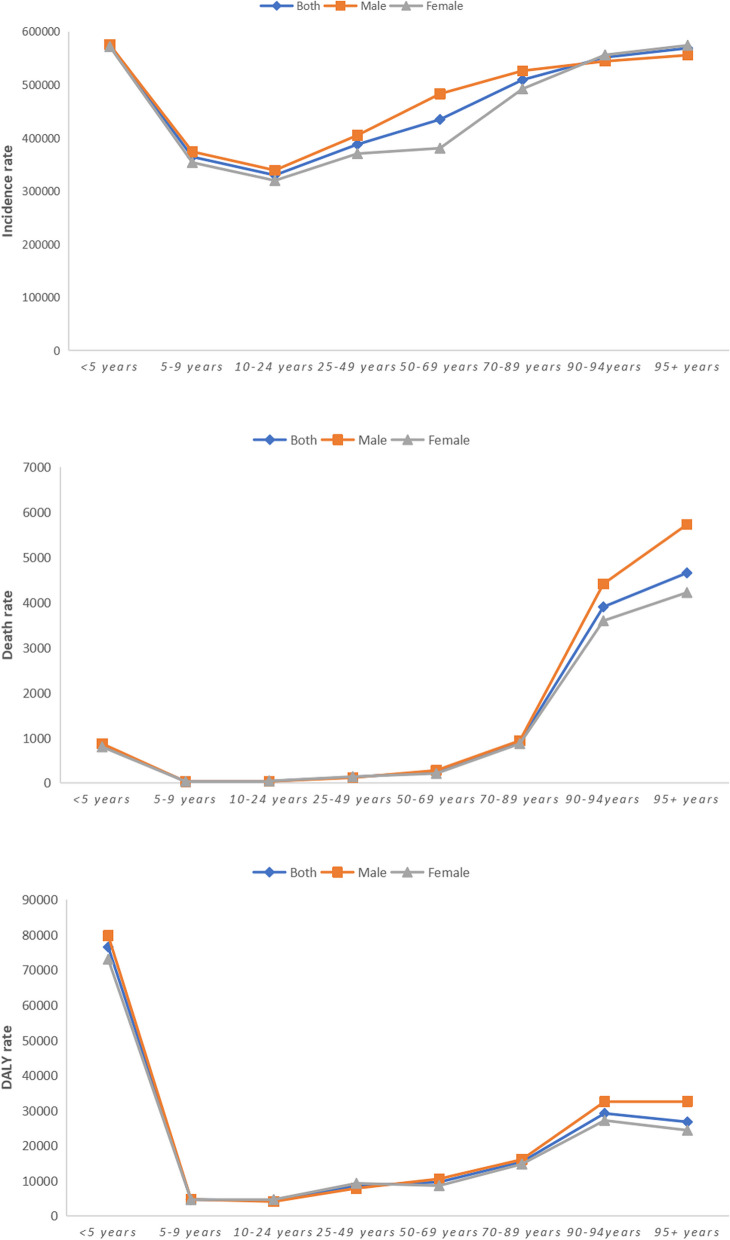
Age-specific incidence, death, and DALY rates of communicable diseases in Pacific Island countries in 2019

The burden of communicable diseases in Pacific Island countries varied widely by age group. The incidence of communicable diseases was highest among children under five years of age, decreased in the 5–24 age group, and then increased with age. Conversely, the death rate of communicable diseases was relatively consistent between the ages of 5 and 69 years but increased after 69 years of age and peaked at 95 + years. DALY rates were highest among children under five years of age, lowest between 5 and 9 years of age, and then fluctuated with increasing age. For more information about the age-specific burden of communicable diseases, refer to Fig. 2.

#### Disease burden by disease type

From 1990 to 2019, the overall disease burden of communicable diseases in Pacific Island Countries declined. However, there was a rapid increase in the disease burden of HIV/AIDS and sexually transmitted infections in these countries. The age-standardized death for HIV/AIDS and sexually transmitted infections increased at an average annual rate of 6.59% and the age-standardized DALY rate increase at an average annual rate of 4.89%.

Respiratory infections and tuberculosis had the highest burden among all diseases, even though their burden decreased compared to 1990. They also had the highest burden of disease among infectious diseases, with the highest standardized morbidity, mortality, and DALY rates of all secondary infectious diseases in Pacific Island countries. For more details, please refer to Table 2.

**Table 2:** Disease-specific incidence, death, and DALY rates of communicable diseases in Pacific Island countries from 1990 to 2019

**Disease category**	**ASIR (/100, 000)**				**ASDR (/100, 000)**				**Age-standardized DALY rate (/100, 000)**			
	1990	2019	AAPC（%）	95% CI	1990	2019	AAPC（%）	95% CI	1990	2019	AAPC（%）	95% CI
Enteric infections	117508	152791	0.935^a^	0.883~0.987	99	54	-2.043^a^	-2.205 ~ -1.881	3359	2019	-1.772^a^	-1.921 ~ -1.623
HIV/AIDS and sexually transmitted infections	14591	14344	-0.052^a^	-0.067 ~ -0.037	6	41	6.589^a^	6.05~7.132	571	2289	4.886^a^	4.552~5.221
Maternal and neonatal disorders	2244	1983	-0.429^a^	-0.450 ~ -0.408	39	32	-0.611^a^	-0.688 ~ -0.534	3272	2817	-0.495^a^	-0.564 ~ -0.427
Respiratory infections and tuberculosis	218163	216183	-0.035^a^	-0.049 ~ -0.021	139	91	-1.431^a^	-1.512 ~ -1.351	6546	4242	-1.488^a^	-1.615 ~ -1.360
Neglected tropical diseases and malaria	19815	11672	-1.322^a^	-2.283 ~ -0.351	19	11	-1.831	-4.141~0.534	1802	1009	-2.341^c^	-4.094 ~ -0.557
Nutritional deficiencies	1785	1675	-0.305^a^	-0.459 ~ -0.151	9	5	-2.153^a^	-2.355 ~ -1.952	922	704	-0.906^a^	-1.05 ~ -0.761
Other infectious diseases	8945	6807	-0.993^c^	-1.450 ~ -0.534	38	18	-2.751^a^	-4.347 ~ -1.130	2836	1296	-2.825^b^	-4.736 ~ -0.876

^a^indicates *P* < 0.001

^b^indicates *P* < 0.01

^c^indicates *P* < 0.05

#### Disease burden by country/region

**Fig. 3:**
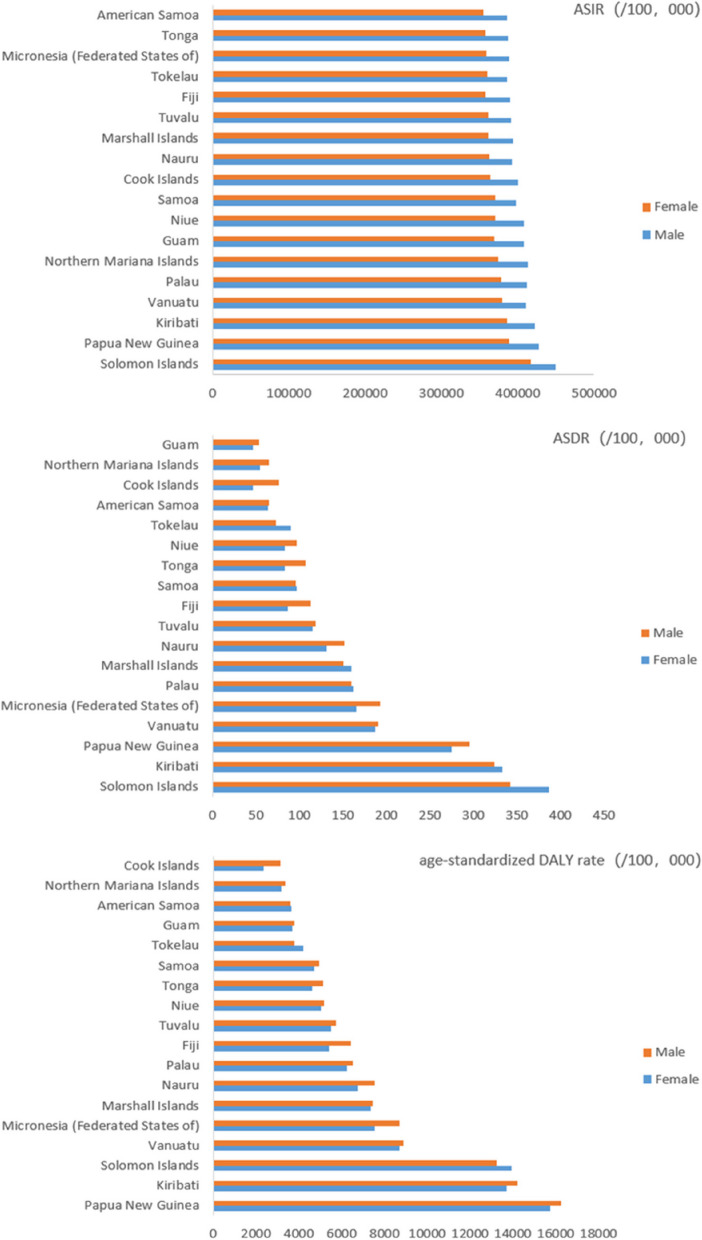
Communicable disease burden status by region in Pacific Island countries, 2019

Among Pacific Island countries, there were substantial regional differences in the disease burden of communicable diseases. The Solomon Islands had the highest burden of disease. As shown in Fig. 3(a), the Solomon Islands had the highest standardized incidence rate (434,102.07/100,000), while Samoa had the lowest (370,393.90/100,000). Figure 3(b) depicts the large variations in standardized mortality rates for infectious diseases by Pacific Island country. The top three standardized mortality rates were observed in the Solomon Islands (363.73/100,000), Kiribati (334.56/100,000), and Papua New Guinea (285.56/100,000), while Guam had the lowest standardized mortality rate (50.42/100,000). Notably, the standardized mortality rate in the Solomon Islands was seven times higher than that in Guam. Figure 3(c) demonstrates the substantial variation in the standardized DALY rates among Pacific Island countries. The top three standardized DALY rates were recorded in Papua New Guinea (16,041.14/100,000), Kiribati (14,017.59/100,000), and the Solomon Islands (13,579.46/100,000). Conversely, the Cook Islands had the lowest standardized DALY rate.

### Risk Factors for communicable diseases in Pacific Island countries

#### Risk factors by disease type

**Fig. 4:**
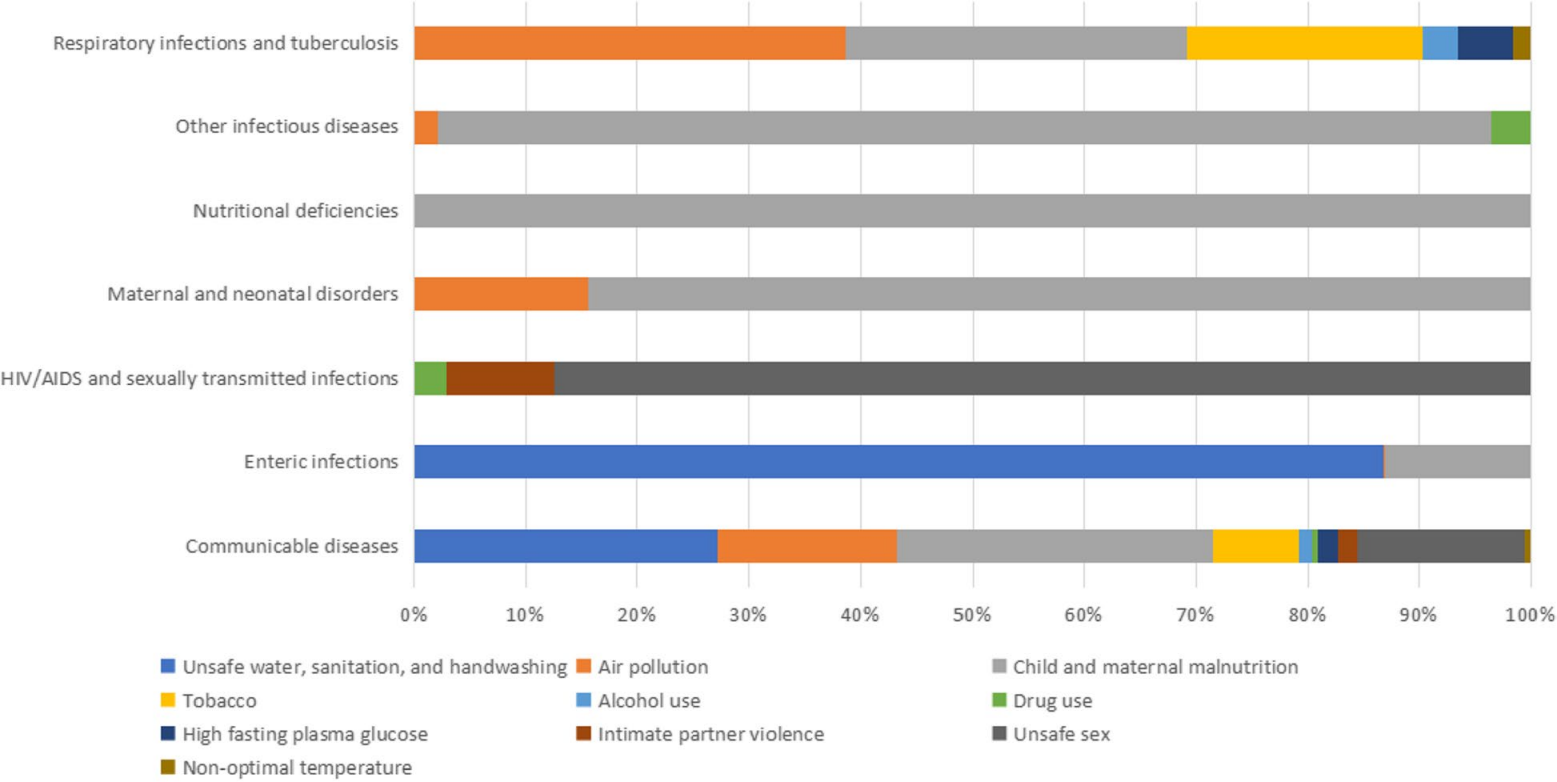
Percentages of deaths caused by level 2 risk factors for communicable diseases in Pacific Island countries in 2019

The main causes of the disease burden of communicable diseases in Pacific Island countries in 2019 were child and maternal malnutrition, which accounted for 28.32% of attributable deaths. This was followed by unsafe water, sanitation, and handwashing (27.14%), air pollution (16.11%), and unsafe sexual (14.96%). Refer to Fig. 4 for detailed information on the distribution of risk factors for all communicable diseases.

For respiratory infections and tuberculosis, the main risk factors attributable to deaths were air pollution (38.63%), followed by child and maternal malnutrition (30.55%). These findings highlight the importance of comprehensive approaches to address environmental health concerns and implement nutrition interventions, which could lead to improve health outcomes in Pacific Island communities.

**Fig. 5:**
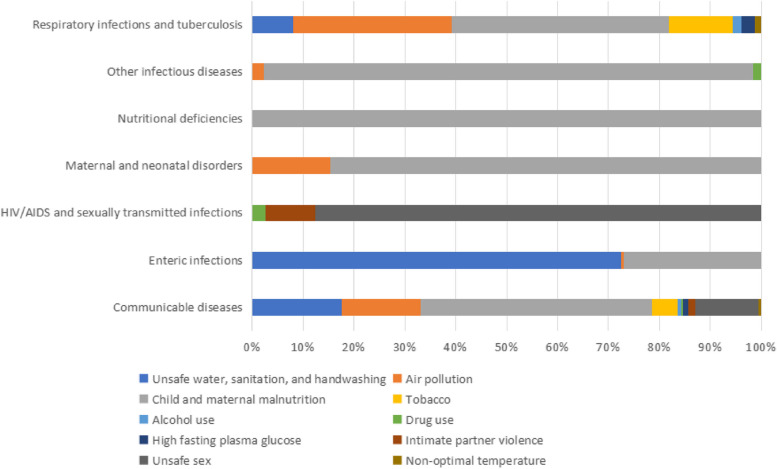
Percentages of DALYs caused by level 2 risk factors for communicable diseases in Pacific island countries in 2019

Child and maternal malnutrition (45.55%) and unsafe water, sanitation, and handwashing (17.58%) were the leading risk factors that accounted for the majority of disability-adjusted life years (DALYs) in Pacific Island countries for communicable diseases in 2019. Air pollution, although slightly lower proportion(15.45%) compared to deaths, continued to be a major risk factor. For more detailed information on the distribution of risk factors for all communicable diseases, refer to Fig. 5.

Child and maternal malnutrition remained the main risk factor responsible for DALYs (42.54%) for respiratory infections and tuberculosis. These findings underscore the importance of addressing malnutrition as a critical risk factor in Pacific Island communities to reduce the burden of communicable diseases.

#### Risk factors by country/region

**Fig. 6:**
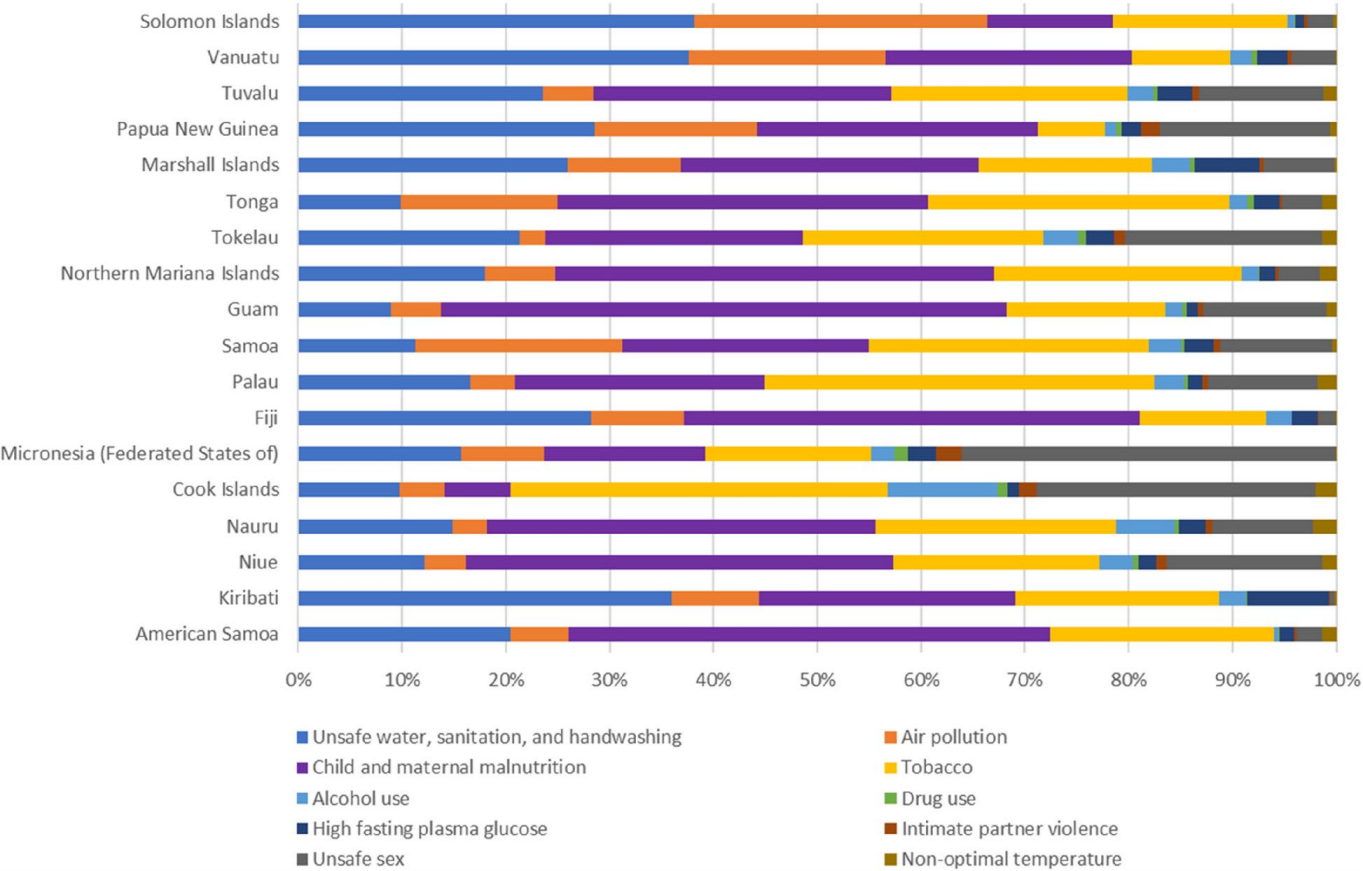
Percentages of deaths attributed to level 2 risk factors for communicable diseases in different Pacific Island countries in 2019

**Fig. 7:**
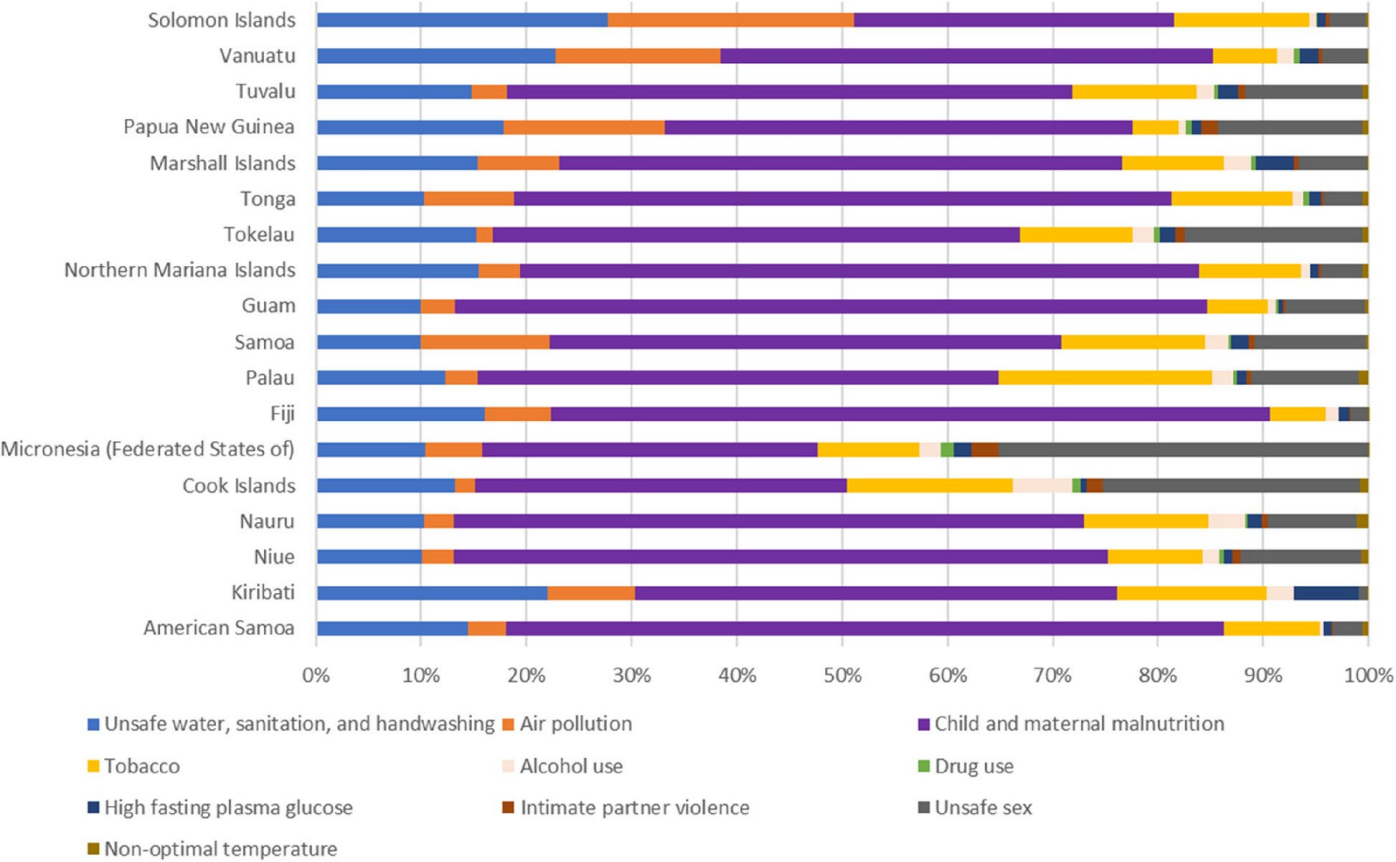
Percentages of DALYs attributed to level 2 risk factors for communicable diseases in different Pacific Island countries in 2019

In 2019, the burden of communicable diseases in Pacific Island countries was influenced by various risk factors that showed considerable geographic differences. Malnutrition in children and pregnant women was a leading cause of death and disability-adjusted life years (DALYs) across all Pacific Island countries. Guam had the highest burden caused by malnutrition in children and pregnant women, accounting for 54.48% of deaths and 71.48% of DALYs, followed by American Samoa, Niue, Fiji, and Northern Mariana Islands. Unsafe water, sanitation, and handwashing, as well as tobacco use, were the two primary risk factors affecting the population in Solomon Islands, accounting for 38.13% of deaths and 28.26% of DALYs. The Federated States of Micronesia, was more substantially affected by unsafe sex intimate partner violence, and drug use compared to other Pacific Island countries. Unsafe sex contributed to more than 35 percent of both deaths and DALYs in the Federated States of Micronesia, which was a substantially higher proportion than in other Pacific Island countries. Additionally, Intimate partner violence and drug use had a far higher impact on the Federated States of Micronesia than on other Pacific Island countries. Refer to Figs. 6 and 7 for more detailed information on the distribution of risk factors by country/region.

## Discussion

The burden of communicable diseases in Pacific Island countries remains high, despite improvements in morbidity and DALYs rate over the past few decades. There were considerable regional differences in the communicable disease burden, with the Solomon Islands being the most affected. Moreover, the burden of communicable diseases was higher for males than for females. Among these diseases, HIV/AIDS and sexually transmitted infections continued to increase rapidly. Additionally, respiratory infections and tuberculosis posed the highest burden among infectious diseases. Furthermore, the Federated States of Micronesia was more affected by unsafe sex, intimate partner violence, and drug use compared to the rest of the Pacific Island countries.

The burden of HIV/AIDS and sexually transmitted infections continues to increase in Pacific Island countries due to several factors. Studies have found that unsafe sexual behaviors, intimate partner violence [18], and drug use are risk factors that contribute to the transmission of HIV. Intimate partner violence is particularly concerning, as it has been shown to increase the risk of women acquiring HIV by 50% [19]. Pacific Island countries face several challenges that make their populations vulnerable to HIV [20]. These include low levels of economic development, poor access to knowledge and services related to HIV testing and treatment, and poverty [21]. Poverty further increases the likelihood of acquiring HIV/AIDS and exacerbates the destructive impact of the disease. Traditional culture and religious beliefs in Pacific Island countries also play a major role in shaping people's awareness and attitudes toward HIV, sexually transmitted infections, and sex education. Many people practice traditional religions that may involve customs and practices that increase the risk of HIV transmission, such as cutting or injections [22, 23]. Therefore, it is essential to expand health education programs that encourage safe sexual practices and reduce the stigma associated with sexual health. A comprehensive approach to health education that includes sex education should be promoted in Pacific Island countries, along with a focus on destigmatizing conversations around sexual health. In conclusion, addressing the rising burden of HIV/AIDS and sexually transmitted infections in Pacific Island countries requires concerted efforts in promoting safe sexual practices, expanding health education programs, reducing stigma around sexual health, and increasing investment in the medical and health sectors.

Respiratory infections and tuberculosis pose a serious disease burden in Pacific Island countries. This differs from South Africa and India, where people have a substantial disease burden of HIV/AIDS, sexually transmitted infections, and diarrhea. The difference in the disease burden is the result of a combination of factors such as underdeveloped medical facilities, high population mobility, low economic levels, monotonous dietary structures, cultural traditions, and more. According to the 2019 Global Health Report, pneumonia and lower respiratory infections are among the deadliest infectious diseases worldwide, ranking among the top four causes of death [4]. Tuberculosis remains the most common cause of death from a single infectious agent. Pacific Island countries experience severe air pollution due to heavy reliance on diesel fuel [24, 25], volcanic eruptions and industrial emissions. Cultural traditions also contribute to this problem. On some islands, poorly ventilated houses, unhygienic dietary habits, and the use of firewood and coal produce harmful gases year-round, contributing to the high incidence of respiratory diseases. Malnutrition further exacerbates the incidence of respiratory infections and tuberculosis. Malnutrition is a persistent challenge in the Pacific Island region. The nutrition of children and pregnant women is critical to health, sustainable development, and progress in middle- and low-income countries and has been included in the global agenda. Therefore, authorities in Pacific Island countries should recognize the synergistic role of nutrition in improving health and development and include nutrition improvement in the health system and program agenda. They should increase the production [26] and distribution of diverse, sustainable, and nutritious foods, provide nutrition education and empowerment, enact policies to reduce the import of high-energy and unhealthy foods, and tailor prenatal care for pregnant women by including nutrition supplements and infectious disease prevention programs [27]. Authorities should vigorously promote delaying marriage and pregnancy [28] and improve the nutritional status of pregnant teenagers.

The burden of communicable diseases was most severe in the Solomon Islands. The country's incidence and death rates rank first among Pacific Island countries, and its DALY rate ranks third. Although the country ranks second in land area and third in population, its economic development level is low, with a per capita GDP ranking second to last [29]. As a result, it is recognized by the United Nations as one of the least developed countries. Compared to other Pacific Island countries, the medical conditions in the Solomon Islands are relatively poor primarily characterized by insufficient healthcare resources [30], poor sanitary conditions [31], a shortage of medical professionals [32], and economic backwardness [33]. Hospitals have rudimentary equipment, shortages of medical staff and medicines are common problems, and medical services in many areas remain limited. Data from the World Health Organization [34] show that the density of medical facilities and personnel in the Solomon Islands is below the global average. The country currently has only nine hospitals, slightly over 900 beds, and a total of 135 clinics and rural health centers across the nation. In addition, the country's tropical climate and environmental conditions make it prone to outbreaks of various communicable diseases, such as malaria and dengue fever. These outbreaks have a serious impact on public health and the health status of the population. The Solomon Islands government needs to increase investment in the medical and health sectors, improve the quantity and quality of medical facilities and personnel, strengthen the coverage and efficiency of medical services, and attract more medical resources and technical support through international cooperation.

The Federated States of Micronesia (FSM) were more affected by three risk factors—unsafe sex, intimate partner violence, and drug use—than many other Pacific Island countries. As a country composed of hundreds of small islands, the geographical location and population distribution of the FSM facilitate an open and diverse social and cultural environment that may also contribute to more cases of unsafe sex and drug use. In some traditional societies, men may view violence against women as a means of exercising power [35], resulting in higher rates of intimate partner violence. The economic and social development of the FSM lags behind that of other countries, with many residents facing poverty and unemployment, which further exacerbate issues such as drug use and unsafe sex. To address these problems, the FSM must implement several measures, such as promoting public awareness and education about unsafe sex and drug use, formulating and implementing stricter laws and policies to prevent and combat intimate partner violence, enhancing social and economic development, improving residents' living standards and health care levels, and strengthening public campaigns for sex education and health education.

## Conclusion

This study analyzed the burden and trends of communicable diseases in Pacific Island countries from 1990 to 2019. The findings provide important insights and emphasize the necessity of targeted public health interventions in the region.

From 1990 to 2019, the standardized incidence rate of communicable diseases in Pacific Island countries showed an upward trend. This indicates a growing burden of these diseases, emphasizing the need for effective prevention and control measures. However, the standardized death rate and standardized DALY rate showed downward trends, suggesting some progress in reducing mortality and disability associated with communicable diseases.

Notably, there were significant differences in the disease burden among different Pacific Island countries. The Solomon Islands had the highest standardized death rate, indicating the urgent need for interventions to address the high mortality caused by communicable diseases in this country. On the other hand, Guam had the lowest standardized death rate, which could serve as a potential model for effective disease control strategies.

The study also identified the main attributable risk factors for communicable disease deaths in Pacific Island countries. Child and maternal malnutrition, unsafe water, sanitation, and handwashing, air pollution, and unsafe sex were the leading risk factors. These findings emphasize the importance of addressing these factors through targeted interventions and policies to reduce the impact of communicable diseases.

However, it is important to acknowledge the limitations of this study. Data availability and quality varied across different Pacific Island countries, which may have affected the accuracy of the estimates. Future research should aim to improve data collection and reporting mechanisms to enhance the validity and reliability of findings.

In conclusion, this study provides valuable insights into the burden and trends of communicable diseases in Pacific Island countries. The identified high-prevalent diseases, such as Respiratory infections and tuberculosis, and countries with the greatest burden, such as the Solomon Islands, require targeted interventions to mitigate the impact of communicable diseases. The findings from this study can inform the development of tailored public health strategies and interventions aimed at reducing the burden of communicable diseases in Pacific Island countries.

### Supplementary Information

**Additional 1: Appendix Table 1.** 18 Pacific island countries. **Appendix Table 2.** Level two risk factors in the GBD

## Data Availability

The data and materials used in this study were obtained from the Global Burden of Disease (GBD) 2019 database. The GBD is a comprehensive and standardized database that contains information on morbidity, mortality, and risk factors for various diseases and injuries worldwide. Access to the GBD data is available through the Institute for Health Metrics and Evaluation (IHME) website (https://www.healthdata.org/gbd). Researchers can request access to the data by registering for a free IHME account and submitting a data request form. The IHME team will review and approve the request based on the research purpose and feasibility. We confirm that all data used in this study were obtained legally and ethically through the appropriate channels and were analyzed in accordance with all relevant guidelines and standards.

## Abbreviations

GBD: Global Burden of Disease
AAPC: Average Annual Percent Change
DALY: Disability adjusted life-year
ASIR: Age standardized incidence rate
ASDR: Age standardized death rate
