# Supplementary material for: Analysis of the burden and trends of communicable diseases in Pacific Island countries from 1990 to 2019
Source: BMC Public Health. 2023 Oct 21;23:2064. doi: 10.1186/s12889-023-16894-z (PMC10590040; doi:10.1186/s12889-023-16894-z)
Supplement: Supplementary file 1 — Additional 1: Appendix Table 1. 18 Pacific island countries. Appendix Table 2. Level two risk factors in the GBD [file 12889_2023_16894_MOESM1_ESM.docx]

**Appendix Table 1** 18 Pacific island countries

| **NO.** | **Country** | **NO.** | **Country** |
| --- | --- | --- | --- |
| 1 | American Samoa | 10 | Guam |
| 2 | Kiribati | 11 | Northern Mariana Islands |
| 3 | Niue | 12 | Tokelau |
| 4 | Nauru | 13 | Tonga |
| 5 | Cook Islands | 14 | Marshall Islands |
| 6 | Micronesia (Federated States of) | 15 | Papua New Guinea |
| 7 | Fiji | 16 | Tuvalu |
| 8 | Palau | 17 | Vanuatu |
| 9 | Samoa | 18 | Solomon Islands |

**Appendix Table 2** Level two risk factors in the GBD

| **No.** | **Risk** | **No.** | **Risk** |
| --- | --- | --- | --- |
| 1 | Unsafe water, sanitation, and handwashing | 11 | Other environmental risks |
| 2 | Air pollution | 12 | Childhood sexual abuse and bullying |
| 3 | Non-optimal temperature | 13 | Occupational risks |
| 4 | Intimate partner violence | 14 | Low physical activity |
| 5 | Unsafe sex | 15 | Dietary risks |
| 6 | Child and maternal malnutrition | 16 | High LDL cholesterol |
| 7 | Tobacco | 17 | High systolic blood pressure |
| 8 | Alcohol use | 18 | High body mass index |
| 9 | Drug use | 19 | Low bone mineral density |
| 10 | High fasting plasma glucose | 20 | Kidney dysfunction |

Note: Numbers 1-10 are in this article refer to risk factors related to communicable diseases in Pacific Island countries.
